# Uterine Balloon Tamponade in Combination with Topical Administration of Tranexamic Acid for Management of Postpartum Hemorrhage

**DOI:** 10.1155/2015/195036

**Published:** 2015-03-12

**Authors:** Masato Kinugasa, Hanako Tamai, Mayu Miyake, Takashi Shimizu

**Affiliations:** ^1^Department of Obstetrics and Gynecology, Amagasaki Co-Op Hospital, 12-16-1 Minamimukonoso, Amagasaki, Hyogo Prefecture 661-0033, Japan; ^2^Shimizu Women's Clinic, 2-2-4 Minamiguchi, Takarazuka, Hyogo Prefecture 665-0011, Japan

## Abstract

While uterine balloon tamponade is an effective modality for control of postpartum hemorrhage, the reported success rates have ranged from the level of 60% to the level of 80%. In unsuccessful cases, more invasive interventions are needed, including hysterectomy as a last resort. We developed a modified tamponade method and applied it to two cases of refractory postpartum hemorrhage after vaginal delivery. The first case was accompanied by uterine myoma and low-lying placenta. After an induced delivery, the patient had excessive hemorrhage due to uterine atony. Despite oxytocin infusion and bimanual uterine compression, the total blood loss was estimated at 2,800 mL or more. The second case was diagnosed as placental abruption complicated by fetal death and severe disseminated intravascular coagulation, subsequently. A profuse hemorrhage continued despite administration of uterotonics, fluid, and blood transfusion. The total blood loss was more than 5,000 mL. In each case, an intrauterine balloon catheter was wrapped in gauze impregnated with tranexamic acid, inserted into the uterus, and inflated sufficiently with sterile water. In this way, mechanical compression by a balloon and a topical antifibrinolytic agent were combined together. This method brought complete hemostasis and no further treatments were needed. Both the women left hospital in stable condition.

## 1. Background

Since postpartum hemorrhage (PPH) is one of the leading causes of maternal mortality worldwide, various strategies have been developed to prevent and control it. World Health Organization, the International Federation of Gynecology and Obstetrics, and the Royal College of Obstetricians and Gynaecologists all recommend a uterine balloon tamponade (UBT) if uterotonics and uterine massage fail to control bleeding [1–3].

Undoubtedly, UBT is an effective treatment option for control of PPH and it has obtained satisfactory results particularly in the management of uterine atony [4]. However, the success rates of current UBT have been reported to be at the level of 60 to 80% [4–8]. In unsuccessful cases, more invasive interventions are needed, including hysterectomy as a last resort. Hence, we consider that the current success rates of UBT still have room for improvement.

A systematic review and meta-analysis indicate strong evidence that intravenous administration of tranexamic acid (TXA) reduces blood transfusion in surgery [9]. It has been also reported that TXA decreases postpartum blood loss after vaginal birth and after caesarean section [10, 11]. Although most of the randomized studies or the cohort studies have suggested no statistically significant increase of thromboembolism with use of TXA, sporadic cases of pulmonary embolism have been reported [11]. The true risk of thromboembolism by TXA remains uncertain because those studies have not statistical powers enough to detect the risks of rare events as pulmonary embolism.

Now, hemostatic effects of topical or intracavitary administration of TXA have been also shown in cardiothoracic or orthopedic surgery [12–14]. Topical use may pose a reduced risk, if any, of thromboembolism because the serum concentration of TXA in topical use would be much lower than systemic use. In obstetrics, however, there is no data on the topical or intracavitary use of TXA.

Based on this background, we speculated that combining both UBT and topical administration of TXA may be useful in cases with severe PPH. Here, we report two cases of refractory PPH in which our modified method of UBT proved effective.

## 2. Case Presentation

### 2.1. Case 1

A 30-year-old multiparous woman was admitted at the 41 weeks of her pregnancy for the purpose of labor induction. It had been pointed out that she had a uterine myoma with a diameter of 7 cm and her placenta was low-lying since 22 weeks of gestation. But the myoma did not obstruct the birth canal, and the placenta was located at least 2 cm apart from the internal uterine os just prior to delivery. With labor induction by oxytocin infusion, she delivered normally a healthy female infant weighing 4,056 g with Apgar scores of 8 and 9 after 1 and 5 minutes.

After delivery of the placenta, excessive hemorrhage occurred and could not be controlled by total 40 units of oxytocin infusion with intravenous fluids, vigorous uterine massage, and bimanual uterine compression. We diagnosed uterine atony after excluding placental retention, cervical or vaginal laceration, and uterine rupture by inspection, palpation, and ultrasound.

The total blood loss was estimated at 2,800 mL or more. Although her consciousness was still clear, her systolic blood pressure temporarily dropped to 80 mmHg and tachycardia (136 bpm) was also observed. The hemoglobin level decreased from 11.0 g/dL before labor to 6.5 g/dL at 2 hours after delivery on laboratory examination. The data on coagulating system including plasma fibrinogen levels (210 mg/dL) were almost normal.

**Figure 1:**
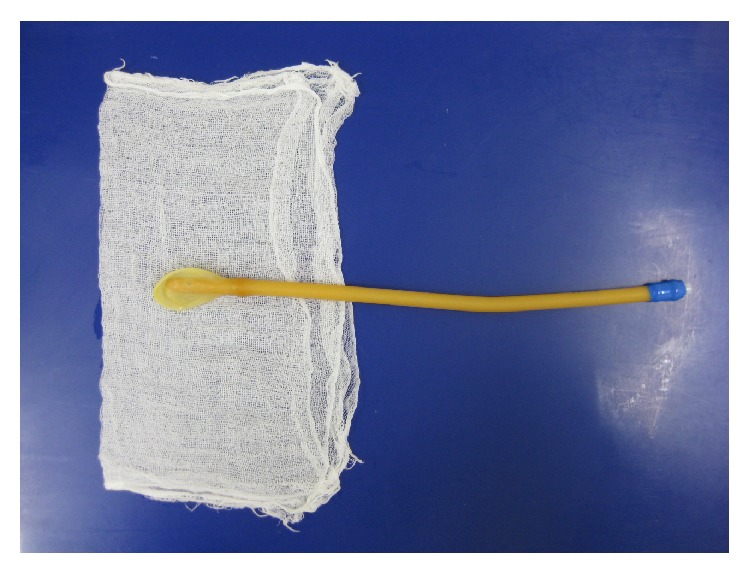
An intrauterine balloon catheter is positioned on the center of two sheets of gauze (30 × 30 cm) which is impregnated with 20 mL of 5% tranexamic acid solution (=1 gram of tranexamic acid) and folded in half.

**Figure 2:**
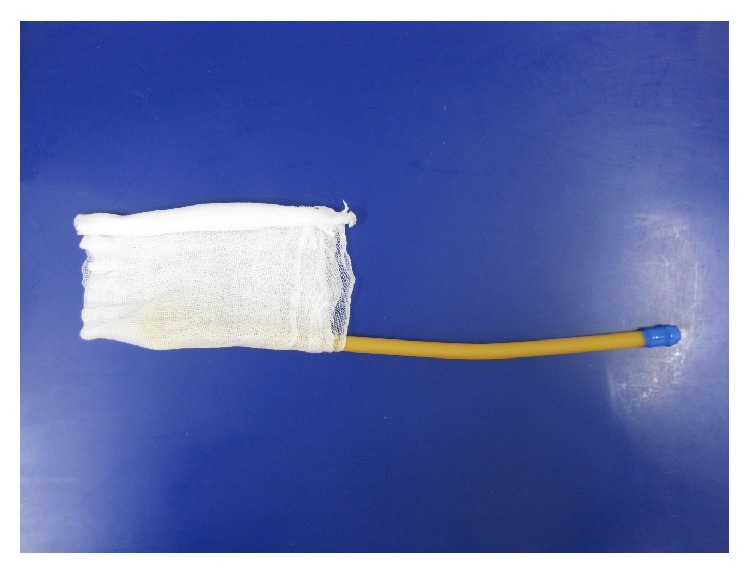
The gauze is further folded in half around the catheter and rolled up from the opposite side toward the catheter.

**Figure 3:**
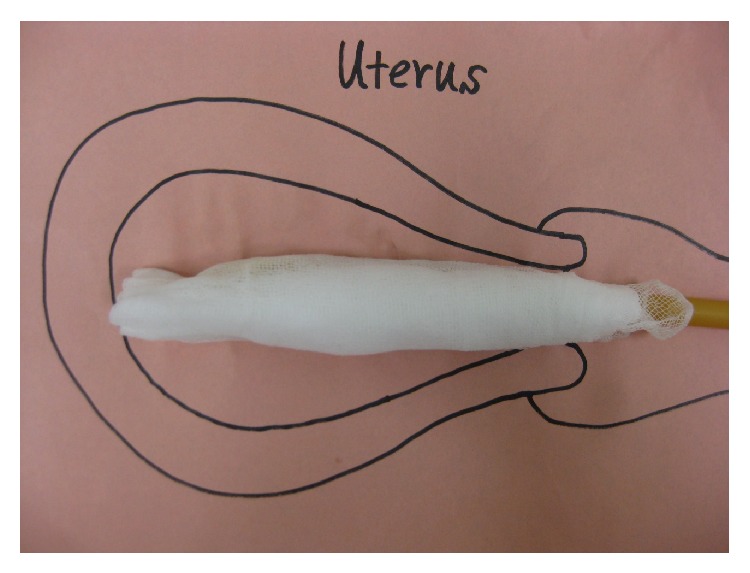
The catheter with the rolled gauze is inserted gently into the uterine cavity using ring forceps. A lubricating jelly may be applied on the tip of the catheter, if needed.

**Figure 4:**
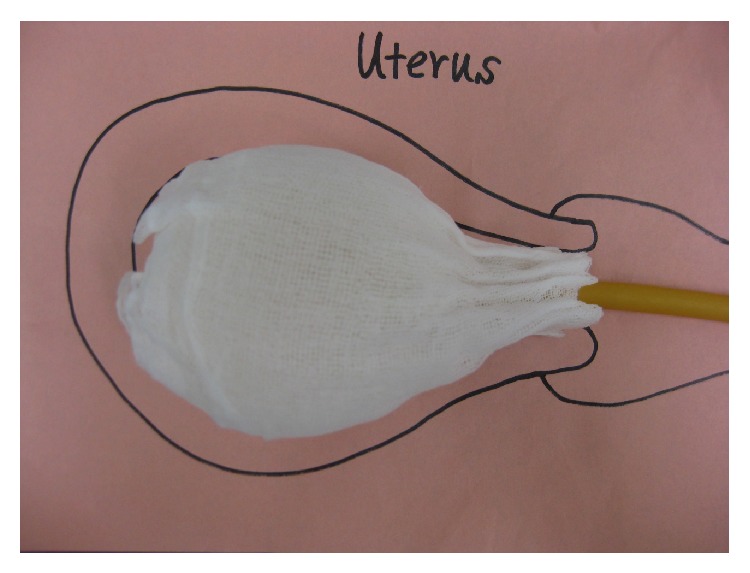
The balloon is inflated with sterile water until it bulges toward the cervix so that it can adequately compress the inner uterus and the tranexamic-acid-soaked gauze can adhere tightly to the bleeding spots. In addition, one-meter long normal gauze is packed in the vagina to prevent the balloon from slipping out.

Then, we tried a modified method of UBT after verbal informed consent of the patient. We used an intrauterine balloon catheter called “Cervical Balloon” (Utsunomiya Seisaku Co., Ltd.), which was produced for the purpose of cervical dilation prior to labor induction. Its balloon part was wrapped in gauze impregnated with 20 mL of 5% tranexamic acid solution (=1 gram of TXA), inserted through the cervix into the uterus and inflated with sterile water (210 mL) until the lowest part of the balloon was fitted just to internal uterine os (Figures 1–4). In addition, a one-meter long normal gauze was packed in the vagina to prevent the balloon from slipping out. A blood transfusion with 6 units each of packed red cells and fresh frozen plasma was performed in parallel. Oxytocin and crystalloid fluid were infused continuously and intravenous antibiotics were given to prevent infection.

Afterwards, the bleeding abated immediately and stopped completely by the next day, when the balloon and gauze were removed after 20 hours of indwelling. She was discharged with her newborn in good condition on the fifth postpartum day.

### 2.2. Case 2

A 32-year-old multiparous woman was referred for abdominal pain, bloody discharge, and an absence of fetal movement at the 37 weeks of her pregnancy. Absence of fetal heartbeat and placental abruption were diagnosed by ultrasound. As labor began spontaneously, we augmented it by amniotomy. The patient delivered a dead female infant weighing 2,928 g, and simultaneously delivered a placenta with large blood clots in a short time. Thereafter, she had profuse hemorrhage refractory to manual compression of the uterus, total 30 units of intravenous oxytocin, and 0.4 mg of methylergometrine.

The laboratory data showed severe anemia and DIC in which the hemoglobin level and the platelet count had dropped from 10.3 to 5.8 g/dL and 72,000 to 36,000/*μ*L, respectively, in 3 hours, and the blood fibrinogen was below the limit of detection. In spite of rapid transfusion with packed red cells and fresh frozen plasma, the bleeding continued and the total blood loss amounted to more than 5,000 mL.

Since she fell into hypotension with systolic blood pressure as low as 69 mmHg and oliguria at that time, we attempted our modified UBT to achieve hemostasis after verbal informed consent. The intrauterine balloon catheter wrapped with TXA-soaked gauze was inserted into the uterus and inflated with 120 mL of sterile water until the lowest part of the balloon was fitted to internal uterine os. The vagina was then packed with gauze. Transfusions amounted to 20 units of packed red cells, 20 units of fresh frozen plasma, and 5 units of platelet concentrates. Uterotonics and antibiotics were also given. The patient was strictly monitored with the balloon in place.

After confirming immediate and complete hemostasis, both the balloon and gauze were removed on the following morning after 10 hours of indwelling. She recovered well and her hematological data also returned to normal. She was discharged in stable condition on the fourth postpartum day.

## 3. Outcome and Follow-Up

Both women were doing well when they visited our hospital for their medical checkup later.

## 4. Discussion

With the intention of reinforcing the effect of UBT, we developed a modified method in which we tried to combine mechanical compression by an inflated balloon and an antifibrinolytic function of topical TXA. This is based on the hypothesis that this combination might be effective in the cases of refractory PPH accompanied with exacerbated fibrinolysis.

Unlike in other fields of surgery, there has been no data on the topical or intracavitary use of TXA in obstetrics, possibly due to technical difficulties in hollow organs with an opening like a uterus. Our method has enabled this, and it is expected to deliver a high concentration of the agent at bleeding spots inside the uterus. Topical use has an advantage over systemic use because the risk of adverse reactions, such as thromboembolism, would be far lower in this route. Wrapping a balloon with gauze fills the gaps between the balloon and the inner uterus and prevents extrusion of the balloon.

Our modified UBT is a minimally invasive, easy to perform, cheap, and fertility-sparing treatment option and it has shown an excellent hemostatic effect even in the setting of DIC (Case 2). Further modifications of this method can be also considered. For example, TXA may be replaced with other hemostatic agents as thrombin, wrapping gauze replaced with sheet-type hemostatic agents as knit oxidized cellulose, or an intrauterine balloon catheter replaced with a set of condom and nelaton catheter in limited resource settings [15].

Our method has a few points to be addressed. The combination of an intrauterine balloon catheter and wet gauze bulks larger than a balloon catheter alone and needs to be inserted into the uterus carefully and gently. Our method can be applied well to PPH after vaginal delivery but may be difficult after cesarean delivery without labor due to the lack of cervical dilation. For such cases, conventional UBT should be indicated first. The wrapping gauze should never be wound up around a catheter simply like a scroll. If that happens, the balloon would fail to expand and would eventually burst. So we recommend the way shown in Figures 1–4.

We used only two sheets of gauze around the balloon catheter, and their ends were visible from the vagina. Therefore, gauze could be easily removed without a fear of retention. The risk of infection can be minimized by removing both the balloon and gauze within 24 hours and administering prophylactic antibiotics.

The catheters we used did not have a drainage channel to monitor bleeding after placement. However, we do not consider it to be essential. Hemostasis can be confirmed in most cases if bleeding through the vagina has stopped promptly after tamponade. Moreover, a drainage channel may be easily occluded by blood clots and repeated flushes may be necessary to avoid it. Instead, serial monitoring of the amount of genital bleeding, vital signs, and the height of the uterine fundus should be continued to perceive ongoing bleeding inside or outside the uterus.

Although our method was successful in two cases of severe PPH, a randomized controlled trial or, at least, a pilot study should ideally be done to prove its effectiveness. However, it is unlikely that such studies can be performed because of rarity of the cases with grave PPH like this report in a small community hospital like ours. Further accumulation of similar cases with the use of our modified UBT will be necessary to evaluate the efficacy and safety of the treatment.

## 5. Learning Points/Take-Home Messages

- We developed a modified uterine balloon tamponade (UBT) in which we combined mechanical compression by an inflated balloon and an antifibrinolytic function of topical tranexamic acid impregnated in wrapping gauze.
- This method was successful in two cases of refractory PPH, one of which was complicated by severe DIC.
- Further accumulation of similar cases with the use of our modified UBT will reveal the efficacy and safety of the treatment.
